# A Competent Antiviral, Antimicrobial, Nontoxic Nanostructured Lipid Carrier System for Safe Use as a Hand Sanitizer: In Vitro and In Vivo Studies

**DOI:** 10.3390/biom16060886

**Published:** 2026-06-16

**Authors:** Eman Samy Shalaby, Mohamed Azab El-Liethy, Sherif Abd-Elmaksoud, Corrado Tagliati, Rawia Mohamed Khalil, Said Ibrahim Shalaby

**Affiliations:** 1 Pharmaceutical Technology Department, Pharmaceutical and Drug Industries Research Institute, National Research Centre, Dokki, Giza 12622, Egypt; rawia_khalil@yahoo.com; 2 Environmental Microbiology Lab., Water Pollution Research Department, Environmental Research and Climate Change Institute, National Research Centre, Dokki, Giza 12622, Egypt; ma.el-liethy@nrc.sci.eg; 3 Environmental Virology Lab., Water Pollution Research Department, Environmental Research and Climate Change Institute, National Research Centre, Dokki, Giza 12622, Egypt; sherifnrc@yahoo.com; 4 AST Ancona, Ospedale di Comunità Maria Montessori di Chiaravalle, Via Fratelli Rosselli 176, 60033 Chiaravalle, Italy; 5 Complementary Medicine Department, Institute of Medical Researches and Clinical Trials, National Research Centre, Dokki, Giza 12622, Egypt; saidshalaby7@gmail.com

**Keywords:** hand sanitizer, alcohol free formulation, antiviral, antimicrobial, nontoxic

## Abstract

Effective hand washing takes time and hand sanitizers that contain alcohol have a number of drawbacks, and frequent use of alcohol may cause skin damage. The objective of this study is to formulate nanostructured lipid carrier systems containing chlorhexidine digluconate to be applied topically for hand hygiene, especially for people sensitive to alcohol. A cytotoxicity experiment was conducted to ascertain the safe dosage for each of the three nano-cream formulas (F1, F2 and F3). Following each treatment, the viral titer was assessed using tissue culture infectious dose_50_ and standard plaque assays. The selected formulation was characterized rheologically. Furthermore, fifteen volunteers of various ages and genders participated in the vivo antimicrobial test of the selected formulation as a hand sanitizer. All of the formulas were found to be safe. Using the disc diffusion method, the three formulations exhibited in vitro antimicrobial effects against different microbes. F1 showed biphasic release, reasonable skin deposition and spherical droplets under a microscope. F1 exhibited a non-Newtonian shear thinning flow behavior. After 30 min, the reduction values for rotavirus and Phix-174 were 21 and 4%, respectively. Additionally, the impact of F1 was assessed on the infectivity of simian rotavirus sa-11 (ds RNA) and Phix-174 (ss DNA) bacteriophage. According to the findings of the in vivo study, the percentage of total bacterial counts that were removed varied from 91 to 100%. Moreover, the range of the removal percentage of total fungi was 95.38 to 100%. In summary, F1 can be used as an economic, safe, and effective hand antiseptic. It can also completely replace alcohol in the market.

## 1. Introduction

In recent decades, microbial infections have become much more common and severe, with high rates of morbidity and mortality, particularly in individuals with impaired immune systems [1]. Antimicrobial resistance can also develop in at-risk patients after receiving prophylactic medication for extended periods of time. According to estimates, microbial infections kill at least 1–5 million people annually and impact 1–2 billion people globally [1,2]. Moreover, the extensive usage of antimicrobial medications is having detrimental effects on antimicrobial therapy since resistant microbial strains are emerging, which results in treatment failures [3]. In addition to resistance-related challenges, antimicrobial medications have a number of drawbacks because of problems with their pharmacokinetic characteristics, activity spectrum, unfavorable side effects, safety profiles, and limited target list [3].

Effective hand washing is time consuming. To speed up this process, hand sanitizers that can be rubbed simply on the skin without the need for rinsing have been developed. They generally involve the use of alcohol as antimicrobial agent [4]. As alcohol evaporates quickly at body temperature it is quick drying. However, there are several disadvantages with alcohol-based hand sanitizers. Alcohol dries the skin. So, its repeated use can lead to skin damage. Alcohol is also highly flammable, which could have dangerous consequences in the event of spills [5]. This necessitates the usage of alcohol-free hand sanitizer. The persistence of antimicrobial agents’ effectiveness in medicine is seriously threatened by bacterial resistance to antibiotics [6]. One of the most popular biocides in antiseptic treatments, hand washing and mouthwash products, and as a disinfectant and preservative, is chlorhexidine digluconate [6]. The antimicrobial mechanism of chlorhexidine involves several sequential steps. Initially, the positively charged chlorhexidine molecules are rapidly attracted to the negatively charged surface of bacterial cells. This is followed by strong and specific adsorption to phosphate-containing components present on the bacterial surface. Chlorhexidine then penetrates the bacterial cell wall, likely through passive diffusion, and interacts with the cytoplasmic membrane, leading to disruption of membrane integrity. Consequently, leakage of low-molecular-weight intracellular constituents, such as potassium ions, occurs along with inhibition of membrane-associated enzymes. In addition, chlorhexidine induces cytoplasmic precipitation through the formation of complexes with phosphorylated intracellular compounds, including adenosine triphosphate (ATP) and nucleic acids. Chlorhexidine is used in hand soaps in concentrations of 2–4% [7]. Chlorhexidine is widely used for the control of dental plaque and gingivitis, as well as for the prevention and treatment of dental caries. It also serves in the management of secondary infections associated with oral surgical procedures, acts as a chemical adjunct during endodontic therapy, and contributes to maintaining the health of peri-implant tissues. Additionally, chlorhexidine is employed for hand hygiene and preoperative skin antisepsis to minimize microbial colonization and reduce the risk of septicemia related to peripheral vascular catheterization, where applicable [8]. Chlorhexidine have been previously reported as to be used in films [8], vaginal inserts [9] and in a device for treatment of periodontal problems [10]. Application of chlorhexidine in a nano formulation to skin would offer a very promising vehicle for drug delivery for hand sanitation for those who have sensitivity to alcohol.

Nanostructured lipid carriers (NLCs) are an advanced generation of solid lipid nanoparticles consisting of a solid lipid matrix incorporated with a specific proportion of liquid lipid. Their distinctive characteristics, including enhanced drug entrapment efficiency, favorable morphological properties, suitability for topical application, and crystalline organization assessed by differential scanning calorimetry (DSC), have been extensively investigated. Furthermore, NLCs maintain a solid-state structure through careful regulation of the liquid lipid concentration within the formulation, thereby enabling controlled drug release behavior. Cosmetic uses for NLCs include cleaning, beautifying, enhancing looks, protecting, and therapeutic functions [11]. Because they are made using methods created in the pharmaceutical industry, NLCs are regarded as pharmaceutical products. Both medicated and unmedicated NLCs are widely used to treat dermatoses and other skin disorders [12]. They include one or more drug compounds that have been dissolved or distributed in an appropriate base. Application of new antimicrobial agents in NLC formulations would be a very promising approach to combat skin fungal infection [13]. Lipid nanoparticles have been extensively investigated as carriers for topical drug delivery to enhance local tissue accumulation, improve drug solubility and bioavailability, and minimize systemic exposure in order to reduce adverse effects. Nanostructured lipid carriers (NLCs) have demonstrated the ability to provide controlled drug release while enhancing skin permeability. In vivo studies have further shown that NLCs facilitate rapid drug penetration through the skin. Moreover, owing to their nanoscale dimensions, typically ranging from 50 to 300 nm, NLCs can be efficiently internalized by cells via endocytosis, thereby improving intracellular drug delivery [14]. Understanding nano–bio interactions is crucial for the rational design, application, and safe use of nanomaterials. Comprehensive characterization of their intrinsic physicochemical properties, including particle size, surface charge, morphology, and surface functionalization, is essential for predicting the behavior, fate, and potential impact of nanomaterials within biological and environmental systems [15].

In the current study we aimed to formulate and evaluate NLCs for topical delivery of chlorhexidine. Drug content and organoleptic property investigations, and cytotoxicity experiments, were utilized to characterize the developed systems. On the selected system, examination of in vitro release and rheology, and photography under a transmission microscope, were carried out. Ex vivo studies including permeation and skin deposition were also investigated. In vivo studies on human volunteers were investigated to uncover new therapeutic strategies to combat bacterial and fungal skin infectious diseases.

## 2. Materials and Methods

### 2.1. Chemicals

BIOCROL^TM^ CG (chlorhexidine digluconate) and NATEROL^®^16 (cetyl alcohol) were gifted from CISME, Italy. Span 60 was obtained from Merck Schuchardt OHG (Hohenbrunn, Germany). Beeswax and Tween 80 were procured from El Gomhuria Company (Giza, Eygpt) for chemicals trade. The MA-104 cell line was purchased from Holding Company for Biological Products & Vaccines VACSERA, Agouza, Giza, Egypt. Dulbecco’s modified Eagle’s medium-high glucose (DMEM) and TrypLE Express were obtained from Gibco^TM^ (Thermofisher Scientific, New York , USA). The 96-well microplate was obtained from Greiner-Bio one, Frickenhausen, Germany, while TSA (Trypticase soy broth) was purchased from BD (Franklin Lakes, NJ, USA). Mueller–Hinton agar was purchased from Thermo Scientific™ Oxoid (Oxoid Limited, Basingstoke, United Kingdom). Molten top agar was obtained from BD, USA.

### 2.2. Preparation of Nanostructured Lipid Carriers

The high-pressure homogenization technique was used to create the pharmaceutical dermal NLCs of the chlorhexidine under study [16,17]. Briefly, the necessary amount of lipid-soluble ingredients—beeswax, span 60, and cetyl alcohol—were combined in a china dish and melted on a hot plate at 80 °C. Chlorhexidine, water and the water-soluble ingredient (Tween 80) were then placed in a different china dish and heated on a hot plate to the same temperature (80 °C). To create NLCs, the melted lipid phase was then combined with the aqueous phase while being continuously stirred at 600 rpm and mixed in a uniform direction. For additional research, the produced formulations (F1, F2, and F3) were kept at room temperature in sterile, tightly sealed containers. The three novel formulations’ physicochemical characteristics and antiviral and antimicrobial assays were examined.

### 2.3. Characterization of the Prepared Nanostructured Lipid Carriers

#### 2.3.1. Drug Content

NLC characterization was evaluated for the all the formulations. One gram of NLC was placed in a 100 mL flask filled with distilled water to determine the drug content. The conical flask was continuously spun for half an hour in order to produce a translucent solution. Whatman filter paper (grade 42) was used to filter the resultant solution, and the filtrate was then gathered. Utilizing a UV spectrophotometer (Shimadzu Corporation, Kyoto, Japan), the absorbance of the resulting dilutions at 295 nm was measured [13], and the following formula was used to determine the amount of chlorhexidine present.

% Drug Content = (Absorbance of sample/absorbance of standard) × 100%
(1)

#### 2.3.2. Cytotoxicity Evaluation by Inverted Light Microscopy Analysis

This test was carried out to assess the possible cytotoxic concentrations of the three formulas (F1, F2 and F3). Ninety-six-well culture microplates were used to prepare MA-104 cells (2 × 10^5^ cells/mL). Starting with a 100 mg/mL solution of dissolved NLC, serial ten-fold dilutions were performed using DMEM as the diluent. Following confluency, 100 μL of each prepared testing formulation concentration was added to the wells to replace the medium. A quantity of 100 μL of DMEM without any sample was utilized as a negative control. For 72 h, the cells were incubated at 37 °C in a humidified environment with 5% CO_2_. At 24, 42, and 72 h following sample incubation, cell morphology was examined under a microscope to assess any possible morphological changes, including loss of confluence, rounding and shrinkage of the cells, cytoplasm granulation, and/or vacuolization [18].

#### 2.3.3. Antimicrobial Activity

##### The Tested Microorganisms and Their Preparation

This study employed seven microbial strains, including four Gram-negative bacteria: *Salmonella typhimurium* (ATCC 14028), *E. coli* (ATCC 25922), *Pseudomonas aeruginosa* (ATCC 10145), and *Pseudomonas putida* (as verified by Biolog GEN III). Moreover, there were one fungal model, *Candida albicans* (ATCC 10231), and two Gram-positive bacteria, *Staphylococcus aureus* (ATCC 43300) and *Listeria monocytogenes* (ATCC 35152). The microbial strains stored at −80 °C were inoculated in 10 mL test tubes containing Trypticase soy broth (TSB). After that, the tubes were incubated at 37 °C with incubation time from one to two days. The grown microbial strains were centrifuged for 20 min at 5000 rpm and washed three times using SDW (sterile distilled water) and the ballets were re-suspended in 5 mL of SDW [19].

##### Disk Diffusion Assay

The disk diffusion method was used to assess the antimicrobial activity of the three formulas (F1, F2, and F3) [19]. Sterile cotton swabs were used to apply the prepared microbial strains on the surface of Mueller–Hinton agar. Then, under aseptic conditions, sterile discs with diameter of 6 mm were individually submerged in formulas (F1, F2, and F3) before being placed on the agar surface of Mueller–Hinton media. For 24 to 48 h, the plates were incubated at 37 °C. An antibiotic disk (FOX 30 mcg) was used as a positive control. Moreover, an SDW disk was used as a negative control. A measuring ruler (HiMedia Co., Maharashtra, India) was used to measure the zone of inhibition (ZI) in millimeters (mm) after the incubation period.

##### Minimal Inhibitory Concentration (MIC)

MIC of the tested sanitizer nanostructured lipid carriers against *E. coli* was assessed. Different concentrations (0.125, 0.250, 0.50, 1, 2, 3, and 4%) were tested. A quantity of 10 μL of *E. coli* was separately added to each concentration. The *E. coli* counts were determined using standard or heterotrophic plate count (SPC) agar according APHA [20]. Non-inoculated SPC agar was used as a negative control. All plates were incubated for 24 h at 37 °C.

##### In Vitro Antiviral Activity

Formulas F1, F2, and F3 were tested for their antiviral properties against the bacteriophage phiX-174 and the rotavirus SA-11. For this, sterile deionized water was used to create nano-cream formulas with a concentration of 100 mg/mL.

##### Viruses’ Propagation Conditions

Prior to rotavirus propagation on MA-104 cells, 10 μg/mL of trypsin was used to activate the virus. To remove cell debris and purify the sample, the rotavirus was centrifuged at 1000× *g* for 5 min. The supernatant, which contained 10^6^–10^7^ TCID_50_/mL, was retained and utilized as a viral stock suspension after being filtered via a 0.2 μm membrane. Then, the suspension was kept at −80 °C until it was needed again. Bacteriophage phiX-174, on the other hand, was grown in an *E. coli* DSM 13127 host in 3.0% TSA with 0.1% glucose, 2 mM CaCl_2_, and 10 mg/mL thiamine for 18 h at 37 °C. Following centrifugation and filtering, the bacteriophage was harvested using the same method previously described for rotavirus.

##### Viruses’ Quantification

Each virus was quantified in cells that were either exposed to it untreated or after being treated with nano-cream formulations. The 50% tissue culture infectious dose (TCID_50_), which is the virus solution concentration at which 50% of the cells exhibit cytopathic effects (CPEs) and is a parameter used to measure and evaluate a virus’s infectivity in cells, was also evaluated during this assay.

To activate the rotavirus SA-11 stock’s infectivity, approximately 10 μg/mL of trypsin was added and left at 37 °C for 30 min. An equivalent volume of the safe concentration of NLCs was combined with a 100 μL aliquot of the activated rotavirus sa-11 at a final concentration of 1 × 10^6^ TCID_50_ per mL. Subsequently, the rotavirus and NLC combinations were incubated for two contact durations of five and thirty minutes at 37 °C. Serial dilutions of rotavirus and NLC rotavirus mixtures were prepared. Then, 96-well tissue culture plates containing monolayers of MA-104 cells at a density of roughly 5.0 × 10^4^ cells per well were filled with aliquots of 100 μL of the 10-fold serial dilutions. To provide adequate assay precision, eight wells were employed for every sample. For seven days, the microplates were incubated at 37 °C with 5% CO_2_. Cells were examined under a microscope every day for CPE during the seven-day incubation period, and the TCID_50_ was calculated. For every sample, this analysis was performed twice. As previously mentioned, equal amounts of viral suspension and each sample were combined, and the mixture was incubated for 5 and 30 min at 37 °C in order to evaluate the impact of the testing solutions on the phiX-174 bacteriophage. Using a conventional plaque assay, the virus titer following each treatment was assessed. The procedure involved adding 0.6% molten top agar to 0.9 mL of phage sample solution and 0.1 mL of *E. coli* culture solution, mixing, and then pouring the mixture onto TSA plates. Following an overnight incubation period at 37 °C, the number of colony-forming units on the plates was counted. Equation (1) was used to determine the difference between the starting (Vi) and final (Vf) viral counts, which represented the virus inhibition following treatment with each sample. This assessment was conducted in duplicate.

(2) $$Virus\;inhibition\;\%=\frac{Vi-Vf}{Vi}\times 100$$

#### 2.3.4. Selection

Although detailed results are presented in Section 3, based on the findings up to Section 2.3.4, F1 was selected from F1–F3 and used for the following evaluations.

#### 2.3.5. Characterization of the Selected Formulations (F1)

##### In Vitro Release Study

In-vitro release of chlorhexidine from the currently selected formulation was carried out by the dialysis membrane diffusion method [21] using a dialysis tubing membrane (molecular weight cut-off, 12,000–14,000 g/mol, Sigma-Aldrich Co, St. Louis, MI, USA). A sample from the selected formulation F1 and from the free chlorhexidine solution equivalent to 100 mg chlorhexidine was introduced in a dialysis bag, previously hydrated with the receptor medium, for 24 h [22]. The dialysis bag was transferred to 100 mL 10% ethanolic phosphate buffer (pH 5.5) (Sirvastava et al. [23]) to fulfil the sink condition, with the temperature kept at 32 ± 0.5 °C, and rotated at 100 rpm [21]. At specified intervals of 1, 2, 3, 4, 5, 6, and 24 h, 5 mL of the sample was taken, and it was replaced with an equivalent volume of new buffer. A UV–visible spectrophotometer (Shimadzu Corporation, Kyoto, Japan) at 260 nm was used. The cumulative percent drug release was then computed. The experiments were performed in triplicate and data are expressed as mean value ±SD. To compare each formulation with the release profile of pure chlorhexidine solution, the cumulative percentage of chlorhexidine released was plotted versus time [24].

##### Organoleptic Characteristics

The selected drug-loaded formulation was evaluated for homogeneity, phase separation, color, texture, and overall physical appearance through visual inspection. Texture and homogeneity were assessed by gently pressing a small amount of the prepared nano-cream between the thumb and index finger. The formulations were examined for consistency and the presence of any coarse particles. In addition, the immediate skin feel, including parameters such as stiffness, grittiness, and greasiness, was also assessed [25].

##### Rheological Measurement

A rheometer with cone and plate test geometry (plate diameter 20 mm, cone angle 4 °C) was used to perform rheological measurement on the chosen formulation. The temperature used for the measurement was 25 ± 0.1 °C. Shear rate was increased from 0 to 200 S^−1^ and then decreased from 200 to 0.15 S^−1^ during 15 S to measure the flow continuously [26].

##### pH Measurement

A digital pH meter (JENWAY 350, Cole-Parmer Ltd., Cambridgeshire, UK) was used to measure the pH after one gram of the chosen formulation was dissolved in 25 milliliters of deionized water. Three separate measurements were taken. Prior to each usage, the pH meter was calibrated using standard buffer solutions (pH 4, 7, and 10).

##### Transmission Electron Microscopy

The morphological characteristics of the selected formulation were investigated to assess structural features, including particle size distribution and shape uniformity. Prior to analysis, the formulation was diluted with bi-distilled water at a dilution ratio of 1:10. A drop of each diluted sample was placed onto a carbon-coated copper grid (300 mesh) and allowed to dry at room temperature. The sample was then negatively stained with 1% phosphotungstic acid before complete drying. Subsequently, the prepared film was examined using transmission electron microscopy (TEM) (JEOL, JEM-1230, Tokyo, Japan) operated at an accelerating voltage of 80 kV at room temperature, and micrographs were captured at appropriate magnifications.

##### Particle Size, Poly Dispersity Index (PDI) and Zeta Potential Measurements

Ten milliliters of distilled water were added to a small amount of nano cream, and the mixture was shaken for 5 min, followed by short-term bath sonication. After that, the sample was placed in a room-temperature quartz cuvette for photon correlation spectroscopy analysis via a Malvern Zetasizer Nano ZS (Malvern Instruments, Malvern, UK) to determine the particle size, PDI, and ZP.

### 2.4. Biological Studies

#### 2.4.1. The Tested Animals

All in vivo biological experiments involving Albino Wistar rats were conducted in accordance with the guidelines of the National Institutes of Health (NIH Publication No. 8023, revised 1978) for the care and use of laboratory animals. The study protocol was approved by the Medical Research Ethics Committee (MREC) of the National Research Centre (NRC), Giza, Egypt (Approval No. 0980223).

In this study, six male Albino Wistar rats aged 9–12 weeks and weighing 180–200 g were obtained from the colony unit of the National Research Centre, Egypt. The animals were maintained under controlled environmental conditions, including a temperature of 23 ± 2 °C, relative humidity of 45–55%, and a 12 h light/dark cycle. The rats were housed in separate hygienic cages with free access to standard pelleted diet and tap water throughout the experimental period.

#### 2.4.2. Ex Vivo Skin Permeation and Skin Deposition

Ex vivo skin penetration and deposition tests of the chosen formulation and chlorhexidine solution were carried out on the dorsal skin of rats. Cervical dislocation was used to kill the rats, and their dorsal shaved skin was then taken and cleansed in double-distilled water. Before being kept at −20 °C until they were used, the integrity of the skins was examined. Vertical Franz diffusion cells were used in permeation research [25]. The skins were placed between the diffusion cell’s donor and receptor compartments, with their stratum corneum facing upward. Following the addition of fresh (10%, *v*/*v*) ethanolic phosphate buffer (pH 7.4), the receptor chamber was maintained at 37 ± 0.5 °C (Ammar et al. [27]) and agitated at 600 rpm [28]. For the formulation, a dosage of 50 mg was applied topically. At predetermined intervals of 1, 2, 3, 4, 6, and 24 h, aliquot samples were extracted from the receptor medium and replaced with an equivalent volume of the new medium. In a spectrophotometric measurement for penetrated chlorhexidine at the designated λmax, the withdrawn samples were contrasted with blanks that had received the same treatment.

All of the research in this test was conducted using the three samples. We were able to ascertain the chlorhexidine penetration profiles from the selected formulation by charting the percentage cumulative amount of chlorhexidine that permeated the skin. Permeation parameters flow (J) and permeability coefficient (Kp) were calculated for each formulation that was the subject of the experiment [29,30]. Plotting the total amount of chlorhexidine penetrated per cm^2^ of rat skin at a steady state versus time allowed for the calculation of the flow (g/cm^2^.h) using linear regression analysis. Using the following equation, the steady state permeability coefficient of chlorhexidine crossing rat skin was calculated:

Kp = J/C
(3)

where C is the amount of chlorhexidine present in the donor compartment and J is the flux.

Following the ex vivo skin permeation experiment, the skins were removed from the diffusion cell and properly cleaned with double-distilled water. After that, skin samples were diced and incubated for 24 h in the refrigerator in 10 milliliters of distilled water. After that, skin samples were sonicated for an hour using the bath sonicator. After the samples were separated by centrifuging them for 10 min at 7000 rpm, the supernatant was filtered using a Millipore syringe filter (0.2 m). Next, using spectrophotometry, the amount of chlorhexidine that was still present in the skin layers was determined at 260 nm in relation to a blank that had not received any treatment [27].

#### 2.4.3. In Vivo Antimicrobial Assay

In vivo antimicrobial study was performed in accordance with relevant guidelines and regulations. Furthermore, all human volunteers or their legal guardian provided their informed consent and agreement, and the prepared nano-cream was applied to the participants’ hands. The National Research Center’s Health Research Ethics Committees in Giza, Egypt approved all experimental procedures (study number: 0980223). Hand swabs (with area 5.0 × 5.0 cm^2^) were taken from 15 volunteers with different ages and sex before and after adding 2 gm of the selected formulation. The collected swab sample was immersed in 5 mL of SDW in first use falcon tubes. The inoculated falcon tube was shaken for 30–60 s to ensure complete microbial release. The microbial counts were determined according APHA [20] as follows: Standard or heterotrophic plate count (SPC) agar was used to estimate the total number of bacteria. For 24 to 48 h, the inoculated plates were incubated at 37 °C. On the other hand, total fungal counts were determined by spreading on the malt extract agar surface. The inoculation fungal plates were incubated from two days to three days at 30 °C. The grown colonies were assessed depending on a colony counter device (Stuart, UK) and the counted colonies were expressed as CFU/cm^2^. The microbial removal was calculated according to Equation (3) before and after F1 addition.

The microbial removal % = Cb − Ca/Cb ×100(4)

where Cb means the total count of the microorganisms before the addition of the selected formulation, while Ca means the total count of the microorganisms after the addition of the selected formulation.

### 2.5. Statistical Analysis

All the obtained results represent replicates and the values were obtained as mean ± SD (standard deviation). The figures were drawn using Excel (Microsoft Office version 10).

## 3. Results

### 3.1. Composition of NLCs

The three prepared NLCs (F1, F2 and F3) were composed of span 60 (3%), beeswax (2%), cetyl alcohol (2.5%) and Tween 80 (0.5%). The water content in F1, F2 and F3 was 88, 89 and 90%, respectively. The amount of chlorhexidine in F1, F2 and F3 was 4, 3 and 2%, respectively (Table 1).

### 3.2. Drug Content

To guarantee the homogeneity of the medication distribution throughout the formulation, drug content uniformity is necessary for semisolid formulations. The % drug content test was carried out to guarantee consistent drug distribution in the produced formulations. Results showed that the percent drug contents of F1, F2 and F3 were 98.12% and 95.12%, and 94.31%, respectively. This indicates that the drug was distributed uniformly throughout the formulations. Therefore, the approach used in this study seems appropriate for nano-cream production.

### 3.3. Cytotoxicity Evaluation

**Figure 1:**
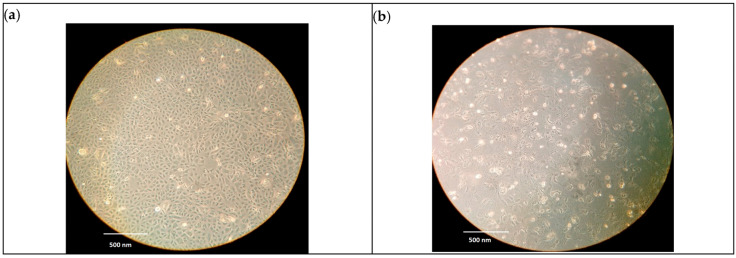
(**a**) Representative inverted light microscopy image of MA-104 cells treated with formulation F1 at 10 mg/mL for 30 min, showing no obvious morphological changes. (**b**) Representative inverted light microscopy image of MA-104 cells treated with formulation F1 at 100 mg/mL for 30 min, showing morphological alterations consistent with cytotoxicity. scale bar = 0.5 μm

The cytotoxicity of the three formulations was first tested using MA-104 cells to confirm that the NLCs alone were safe and nontoxic. Serial dilution of prepared NLC concentrations was applied to the cells and their morphology was continuously evaluated by an inverted light microscope. According to the results obtained through this test (Figure 1), a 10 mg/mL concentration of NLC formulas is safe and no cytotoxic effects such as cell rounding and shrinking, loss of confluence, or cytoplasm granulation were observed in the tested cells up to 72 h (Table 2).

### 3.4. Antimicrobial Assay

**Figure 2:**
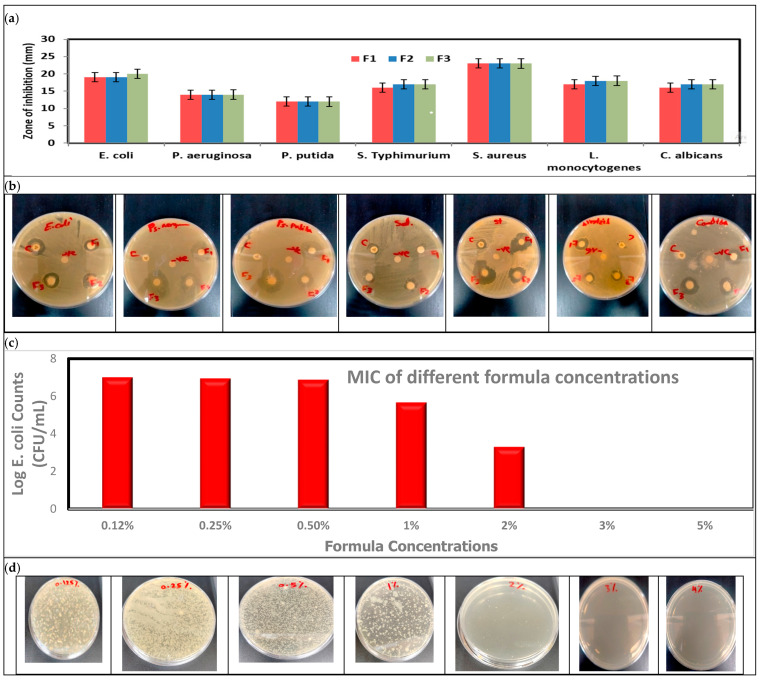
(**a**) The zone of inhibition (ZI) diameters of the tested sanitizer nanostructured lipid carriers against seven microbial strains. (**b**) Plates illustrate ZI of the tested sanitizer nanostructured lipid carriers against seven microbial strains, in addition to using Fox antibiotic disk as a positive control and saturated disk with sterile distilled water (SDW) as a negative control. (**c**) Figure shows the minimal inhibitory concentration (MIC) of the tested sanitizer nanostructured lipid carriers against *E. coli*. (**d**) *E. coli* colonies on plates using different concentrations (0.125, 0.25, 0.5, 1, 2, 3, 4%) of the tested sanitizer nanostructured lipid carriers.

The inhibition zone diameters of F1 against *E. coli*, *P. aeruginosa*, *P. putida*, *S. typhimurium*, *Staph aureus*, *L. monocytogenes* and *C. albicans* were 19, 14, 12, 16, 23, 17 and 16 mm, respectively. The inhibition zone diameters of F2 against *E. coli*, *P. aeruginosa*, *P. putida*, *S. typhimurium*, *Staph. aureus*, *L. monocytogenes* and *C. albicans* were 19, 14, 12, 17, 23, 18 and 17 mm, respectively. Moreover, the inhibition zone diameters of F3 against *E. coli*, *P. aeruginosa*, *P. putida*, *S. typhimurium*, *Staph. aureus*, *L. monocytogenes* and *C. albicans* were 20, 14, 12, 17, 23, 18 and 17 mm, respectively (Figure 2a,b). The results showed that the three prepared formulas (F1, F2 and F3) have strong antimicrobial effect against *E. coli*, *P. aeruginosa*, *P. putida*, *S. typhimurium*, *Staph. aureus*, *L. monocytogenes* and *C. albicans*. The results of the minimal inhibitory concentration (MIC) of the tested sanitizer nanostructured lipid carriers against *E. coli* showed that the MIC was 2% at which more than three log_10_ has been removed. In addition to this, complete removal of *E. coli* was shown with 3% and 4% (Figure 2c,d).

### 3.5. Antiviral Activity

The F1, F2, and F3 NLCs showed no antiviral activity against the rotavirus (SA-11) and phiX-174 viruses after five minutes of exposure. However, after 30 min contact time, F1 showed 21 and 4% reduction in rotavirus and Phix-174 infection, respectively, while F2 and F3 did not show any antiviral activity (Table 3 and Table 4).

### 3.6. Selection of the Optimum Formulation

All the formulations showed antibacterial activity and were able to encapsulate the drug. However, the formulation F1 was the only one that showed antiviral activity against Phix-174 and rotaviruses, so it was selected for further evaluations.

### 3.7. Characterization of the Prepared NLCs

#### 3.7.1. Organoleptic Properties

Table 5 lists the chosen topical formulation’s organoleptic characteristics, such as its physical appearance, color, texture, phase separation, homogeneity, and instant skin sensation. The NLC’s smooth texture and attractive look were demonstrated by the results, which also revealed that there were no indications of phase separation and that all of the ingredients were homogenous. Every formulation had a white hue. The current results are comparable to those that have been previously reported [25].

#### 3.7.2. In Vitro Release Study

**Figure 3:**
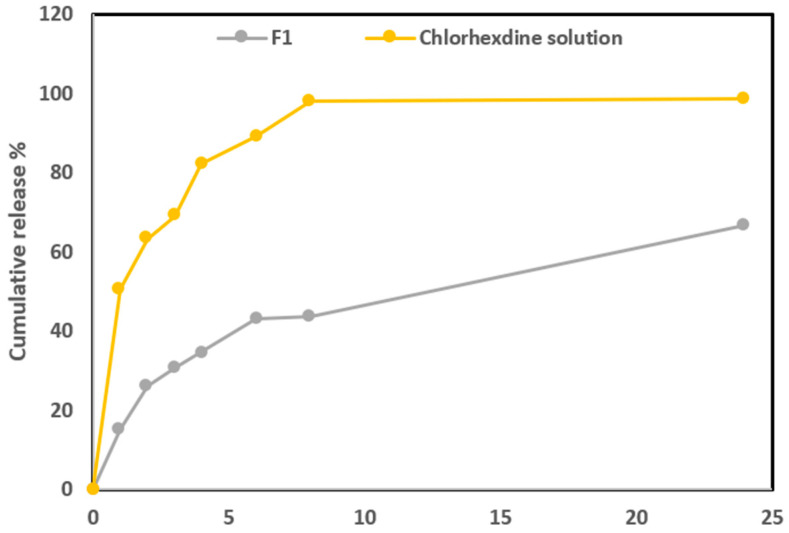
In vitro release of the selected formula F1 and the drug solution.

Preliminary experiments conducted in this study demonstrated that chlorhexidine solution exhibited a faster release profile compared with the nanostructured lipid carriers (NLCs) (Figure 3). The slower drug release from NLCs could be attributed to the presence of the lipid matrix, which acts as a barrier limiting the diffusion of chlorhexidine from the nanoparticles. In contrast, the enhanced cumulative dissolution observed with NLCs was likely associated with the disordered crystalline structure of the encapsulated drug, which improved its solubilization and supported its suitability for nanoparticle formulation.

#### 3.7.3. Rheology of NLCs F1

**Figure 4:**
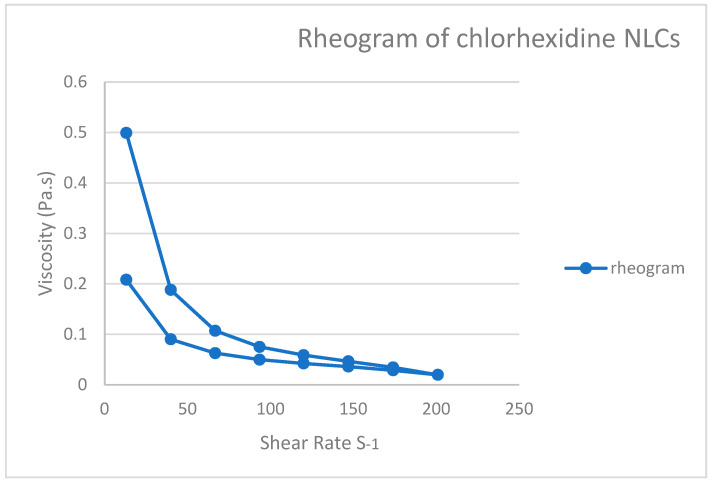
Rheogram of the selected F1 formulation.

The rheological data of the formulation F1 are illustrated in Figure 4. The viscosity values of the prepared formulation at 25 ± 0.5 °C, simulating room temperature, ranged between 0.449 and 0.0197 Pa.s. On the other hand, Figure 4 reveals viscosity curves (viscosity versus shear rate) of the selected nano-cream formulation. The formulation exhibits a shear rate-dependent change in viscosity, with a steady drop in viscosity as the shear rate increased. This suggests that the flow behavior of chlorhexidine NLCs is non-Newtonian and shear-thinning (pseudoplastic).

#### 3.7.4. pH Measurement

In most cases, pH was determined by means of a flat glass electrode. The pH value of the selected formulation was 5.56.

#### 3.7.5. Transmission Electron Microscope

**Figure 5:**
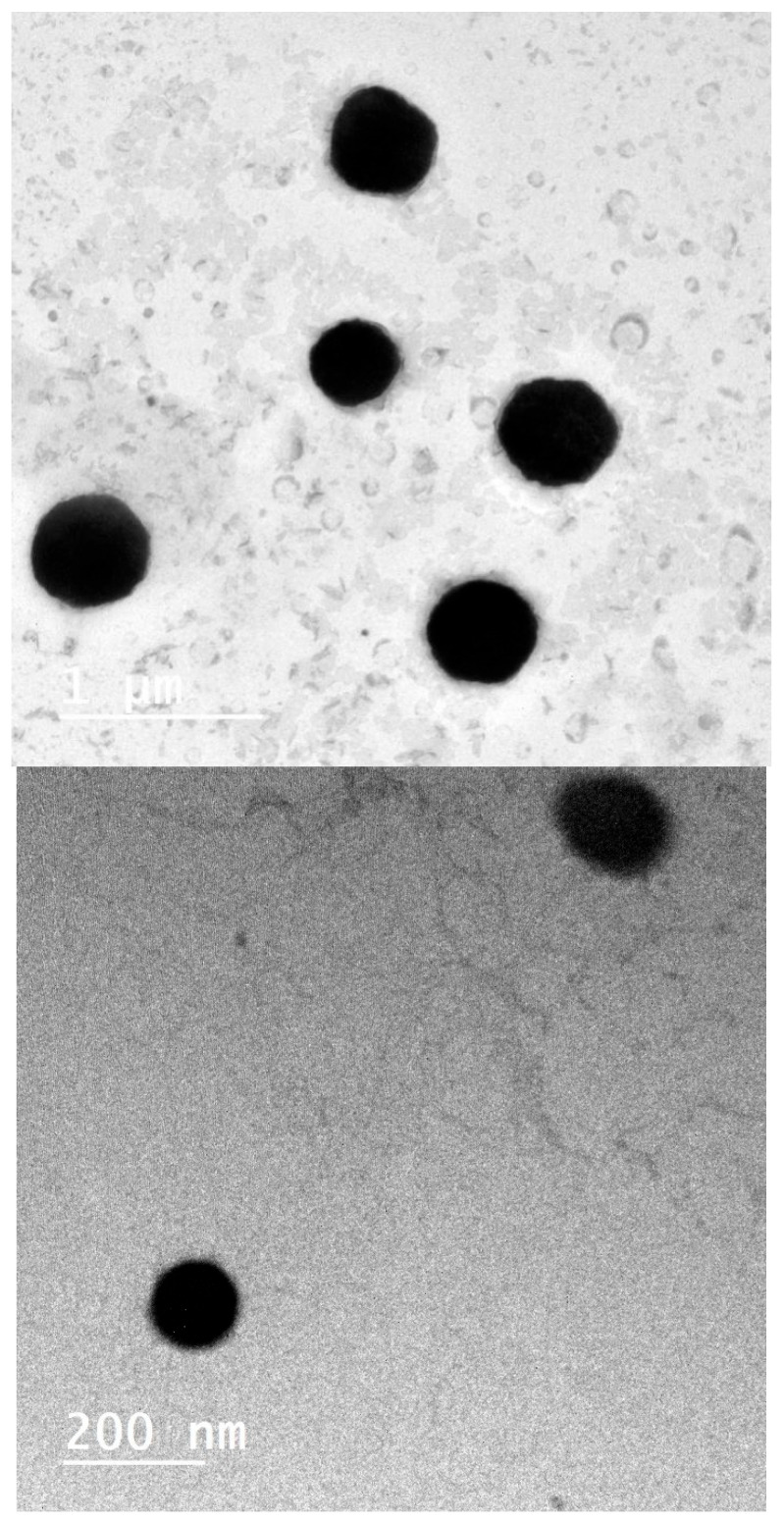
Transmission electron microscopy imaging of the selected formulation F1.

As illustrated in Figure 5, chlorhexidine was successfully incorporated into formulation F1, and the prepared nanostructured lipid carriers (NLCs) exhibited a well-defined spherical morphology with intact structural integrity. A slight variation was observed between the particle sizes obtained by transmission electron microscopy (TEM) and dynamic light scattering (DLS). This discrepancy may be attributed to the different measurement conditions employed by the two techniques. TEM determines particle size in the dry solid state after sample preparation and drying, which may lead to particle flattening and an apparent increase in size. In contrast, DLS measures the hydrodynamic diameter of nanoparticles dispersed in aqueous media, where the presence of a solvation layer contributes to the observed particle size.

#### 3.7.6. Particle Size, PDI and Zeta Potential Measurements

The formulation F1 particle size was found to be 533.4 ± 0.134 nm, and PDI was 0.324. Zeta potential was −34 ± 1.13 mV. The particle size values fall within the optimal nanoscale range for cellular uptake. This is consistent with previous studies that reported sizes around 500 nm for efficient topical delivery systems, suggesting good potential for biological interaction. These values mean that the formulations are physically stable, homogenous and have a particle size suitable for topical applications. Particle size was 220 nm.

### 3.8. In Vivo Studies

#### 3.8.1. Ex Vivo Permeation and Skin Deposition

**Figure 6:**
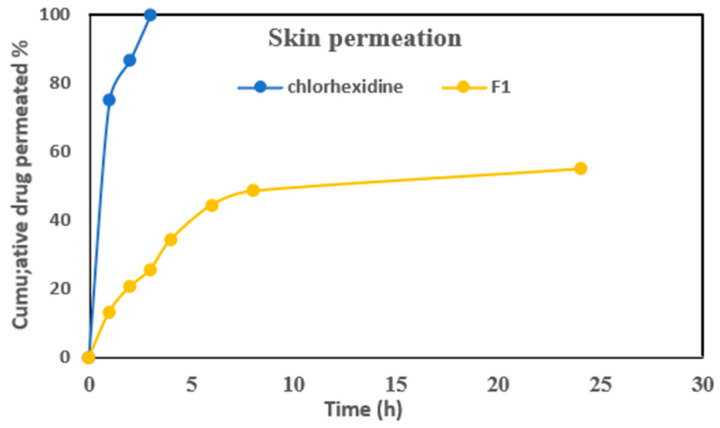
Permeation profile of F1 and chlorhexidine solution across rat skin.

**Figure 7:**
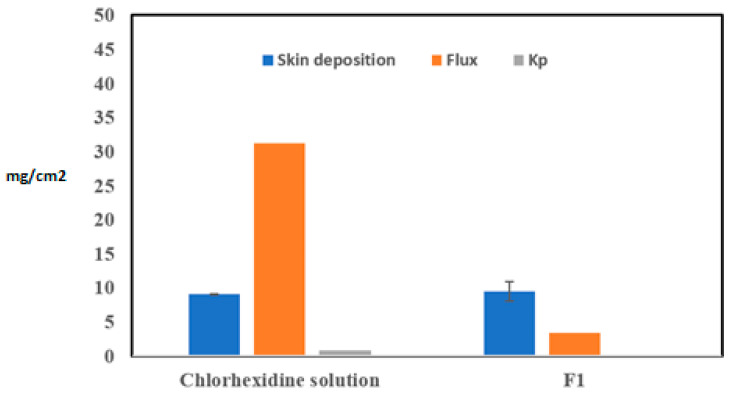
Skin deposition of the selected chlorhexidine NLCs, flux and permeability coefficient (Kp).

The ex vivo skin permeation results are illustrated in Figure 6; chlorhexidine solution showed the highest permeation (reaching 100% after only 2 h) (*p* < 0.05) and flux values, followed by chlorhexidine nano-cream formulation. Flux and Kp are introduced in Figure 7. Regarding skin deposition values, the highest value was found for F1, followed by chlorhexidine solution (Figure 6). Skin deposition of F1 was found to be 2.47 ± 0.24 mg, significantly higher (*p* < 0.05) than that of free chlorhexidine solution (1.77 ± 0.23 mg). A cluster of corneal cells encourages accumulation over a number of hours. Because of this, extremely hydrophilic drugs that are encased in NLCs are believed to have a “reservoir” in the stratum corneum that permits their constant release into the skin’s deeper layers.

#### 3.8.2. In Vivo Antimicrobial Activity

**Figure 8:**
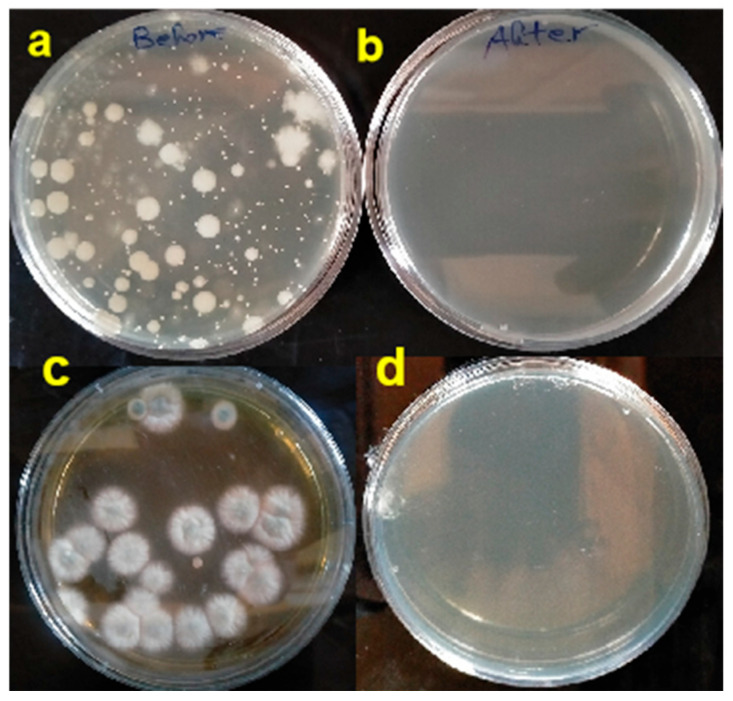
In vivo antimicrobial study of the application of F1 applied as a hand sanitizer on human hands. Total bacteria before adding F1 (with bacterial colonies appearing) (**a**), and after adding F1 (with an absence of bacterial colonies) (**b**). Total fungi before adding F1 (with fungal colonies appearing) (**c**), and after adding F1 (with an absence of fungal colonies) (**d**).

To assess the prepared nano-cream F1’s in vivo antimicrobial capacity, the hands of fifteen volunteers were inspected for the presence and total number of microorganisms, including bacteria and fungi, both before and after F1 was applied. In this study, the ages of volunteers ranged between 6 and 65 years old, with 66.6% male. Moreover, bacteria and fungi were detected in 100 of the samples (80%) of the collected samples prior to adding F1. Prior to the use of F1, the highest values for total bacterial and fungal counts were 6200 and 3500 CFU/cm^2^, respectively (Table 6). After F1 application, the removal percent of bacteria ranged between 91 and 100%. In addition to this, complete removal (100%) of bacteria was observed in 8 out of 15 samples (53.3%). The fungal removal percent ranged between 65.38 and 100% (Table 6). The results showed that the prepared NLCs (F1) exhibited potent in vivo antimicrobial activity against the examined fungus and bacteria, facilitating the effective removal of these microorganisms from the hands of the volunteers (Figure 8).

## 4. Discussion

The hands are the main site of transmission for infections caused by microbes because they can harbor both pathogenic microorganisms and healthy microbiota. The WHO has recommended maintaining proper hygiene, especially with regard to hand hygiene, since SARS-CoV-2 has endangered lives worldwide [31]. Therefore, in this study, antimicrobial activities of the three prepared NLCs against seven microbial pathogens have been investigated. Bonez et al. [32] indicated that chlorhexidine has antimicrobial activity against bacteria and fungi. Chlorohexidine has been shown in numerous studies to have virucidal properties against a variety of viruses, including Severe Acute Respiratory Syndrome-Coronavirus (SARS-CoV) [33], Influenza A (InfluA), and Herpes Simplex Type-1 (HSV-1) [34]. According to Karpiński and Szkaradkiewicz [35], Baqui et al. [36], and Xu et al. [37] the mechanisms for virucidal effect of chlorohexidine are the deterioration of nucleotide carbon chains and the impact on the inactivation and/or blocking of viral proteins, which may explain the efficacy against both viruses tested in the present study. Both rotavirus and the bacteriophage phiX-174 are non-enveloped viruses. Many investigations have shown that chlorohexidine is more efficient against lipid-enveloped microorganisms (Bernstein et al. [38]), which supports the reduced activity of chlorohexidine shown in this study for both viruses.

Cytotoxicity was assessed qualitatively by inverted light microscopy to identify a safe concentration range for subsequent experiments. This approach does not replace quantitative viability assays; therefore, additional studies using MTT or similar methods are warranted to validate these observations.

F1 showed a significantly (*p* < 0.05) lower release % than chlorhexidine solution. This may be due to the significant (*p* < 0.05) high viscosity of NLC formulations than chlorhexidine solution. Similar results were previously reported by Vieira et al. [39]. For every formulation, the drug release was seen to happen in two stages: a burst release that lasted for the first hour and a regulated release phase that lasted for 24 h. The initial burst release phase of the developed formula was significantly smaller (*p* < 0.05) with F1 compared to the drug solution; this may be explained by the low drug concentration on the system’s surface, which resulted in a shorter initial release phase. A sustained release pattern that generally developed and prolonged a controlled release method followed a slightly limited drug release during the second phase. For topical treatment, both burst and sustained release are crucial characteristics. Burst release enhances the uptake of pharmaceutical substances, offers quick relief, and has a quick commencement of action. Sustained release comes next, lowering the frequency of administration, preserving a long-lasting medication level, and producing effective therapeutic results [38].

When determining whether a medication system is suitable for topical application, the rheological behavior of semisolid formulations is a crucial consideration [29]. This dependent change in viscosity is a desirable characteristic since it makes it easier to apply and disseminate the preparation on the skin and permits removal from the container [1]. The preparation’s network structure disintegrates when a shear force is applied, gradually reducing its viscosity [40]. pH matches the pH of normal healthy skin.

Strong antimicrobial activity was observed in two in-house-developed herbal-alcohol-based hand sanitizers that contained *Azadirachta indica*, *Citrus limon*, *Zingiber officinale*, and *Aloe vera* (HS1), with *Ocimum sanctum* (HS2) substituting for *Zingiber officinale*, according to an in vivo study conducted by Tulsawani et al. [31] on forty volunteers. They also discovered that HS1 and HS2 outperformed WHO sanitizers with an alcohol base in terms of effectiveness.

All of the volunteers in this study showed no symptoms of infection, trauma, dermal abrasion, or irritation in or around the formulation application area. Furthermore, no potential adverse effects were reported by the volunteers after the formulation was applied.

## 5. Conclusions

The objective of this study was to formulate topical NLCs containing chlorhexidine as nontoxic, potent, cost-effective and antimicrobial NLCs can be used as hand sanitizer. Three formulas of chlorhexidine (4% (F1), 3% (F2) and 2% (F3)) were prepared and characterized. The results showed the three formulations have no cytotoxicity and have strong in vitro antimicrobial effects against *P. aeruginosa*, *P. putida*, *E. coli*, *S. typhimurium*, *Staph. aureus*, *L. monocytogenes* and *C. albicans.* Moreover, the three formulations have antiviral activity against rotavirus and bacteriophage phiX-174. The characterization results of F1 showed that it has a white color, smooth texture, and pH of 5.56, with a viscosity range between 0.449 and 0.0197 Pa.s. The formulation F1 showed biphasic release, reasonable skin deposition, and spherical droplets under a microscope. By applying F1 as a sanitizer on human hands of 15 volunteers, the removal of bacteria ranged between 91 and 100%, while the reduction in fungi ranged between 65.38 and 100%. Skin irritation is a common side effect of commercially available alcohol gel-based disinfectants. The ingredients in this skin nano-cream composition improve the microbiological quality of cosmetics while simultaneously increasing the softness and moisture content of the skin.

## Figures and Tables

**Table 1:** Composition of chlorhexidine NLCs.

Composition	The Three Prepared Formula		
	F1 (%)	F2 (%)	F3 (%)
Span 60	3	3	3
Beeswax	2	2	2
Cetyl alcohol	2.5	2.5	2.5
Tween 80	0.5	0.5	0.5
Water	88	89	90
Chlorhexidine	4	3	2

**Table 2:** Cytotoxicity of tested NLC formulations on MA-104 cells.

Tested Formulation	Concentration of Nanostructured Lipid Carriers	
	100 mg/mL	10 mg/mL
F1	Toxic	Safe
F2	Toxic	Safe
F3	Toxic	Safe

**Table 3:** The antiviral activity of three nano-cream formulations against Rotavirus (SA-11).

Sample	Nanostructured Lipid Carrier Concentration	Viral Counts (TCID_50_ ^†^/mL) ±SD°	Reduction % ±SD
Initial count	10 mg/mL	3.98 × 10^5^ ± 2.8 × 10^3^	N.A.
F1	10 mg/mL	3.16 × 10^5^ ± 2.8 × 10^3^	21 ± 0.007%
F2	10 mg/mL	3.98 × 10^5^	0%
F3	10 mg/mL	3.98 × 10^5^	0%

^†^ TCID, tissue culture infectious dose; SD°, standard deviation; N.A., not applicable.

**Table 4:** The antiviral activity of three NLC formulations against Phix-174 virus.

Sample	Nanostructured Lipid Carrier Concentration	Viral Counts (PFU ^†^/mL) ±SD°	Reduction % ±SD
Initial count	10 mg/mL	4.70 × 10^5^	N.A.*
F1	10 mg/mL	4.50 × 10^5^ ± 2.8 × 10^4^	4 ± 1.4%
F2	10 mg/mL	4.70 × 10^5^	0%
F3	10 mg/mL	4.70 × 10^5^	0%

^†^ PFU, plaque forming unit; SD°, standard deviation; * N.A., not applicable.

**Table 5:** Organoleptic properties of F1.

Formulation	Physical Appearance	Color	Texture	Phase Separation	Homogeneity	Immediate Skin Feeling
F1	Opaque	White	Smooth	No	Homogenous	Moisturizing, light, no grittiness, not greasy

**Table 6:** The overall number of bacteria and fungi in the swab samples taken from people’s hands before and after applying F1.

No	Age	Gender *	Adding NLCs	CFU/cm^2^			
				Total Bacteria		Total Fungi	
				Counts	R% ^†^	Counts	R%
1	65	M	Before	1800	-	10	
			Data	66	96.33%	0.0	100%
2	38	F	Before	190		20	
			Data	10	94.73%	0.0	100%
3	35	M	Before	2100		0	
			After	12	99.52%	0	-
4	42	M	Before	1500		0	
			After	10	99.33%	0	-
5	41	M	Before	8200		12	
			After	41	99.50%	0.0	100%
6	34	F	Before	85		200	
			After	0.0	100%	8	96.0%
7	38	M	Before	70		200	
			After	0.0	100%	8	96.0%
8	29	M	Before	500		3500	
			After	45	91.0%	80	97.71%
9	45	M	Before	90		130	
			After	0.0	100%	6	95.38%
10	40	M	Before	6200		30	
			After	0.0	100%	0.0	100%
11	15	F	Before	100		90	
			After	0.0	100%	0.0	100%
12	12	M	Before	80		10	
			After	0.0	100%	0.0	100%
13	6	F	Before	50		50	
			After	0.0	100%	0.0	100%
14	40	F	Before	200		45	
			After	0.0	100%	0.0	100%
15	34	M	Before	1000		40	
			After	20	98%	0.0	100%

* M, male; F, female; R% ^†^, removal percent.

## Data Availability

The original contributions presented in this study are included in the article. Further inquiries can be directed to the corresponding author.

## Abbreviations

The following abbreviations are used in this manuscript:

NLC	Nanostructured lipid carrier
CFU	Colony-forming unit
DMEM	Dulbecco’s modified Eagle’s medium-high glucose
SD	Standard deviation
SDW	Sterile distilled water
TCID	Tissue culture infectious dose
Vf	Final virus count
Vi	Starting virus count
ZI	Zone of inhibition
